# Editorial: Metabolic engineering and synthetic biology for sustainable microbial cell factories

**DOI:** 10.3389/fmicb.2026.1871007

**Published:** 2026-07-08

**Authors:** Mugesh Sankaranarayanan, Karthik Loganathan, Lorenzo Pasotti

**Affiliations:** 1 Centre for Metabolic Engineering and Synthetic Biology, Department of Biotechnology, Vel Tech Rangarajan Dr. Sagunthala R&D Institute of Science and Technology, Avadi/Chennai, Tamil Nadu, India; 2 Arqgene, Vellore, Tamil Nadu, India; 3 Laboratory of Bioinformatics, Mathematical Modelling and Synthetic Biology, Department of Electrical, Computer and Biomedical Engineering, University of Pavia, Pavia, Italy

**Keywords:** bioactive compounds, genome mining, metabolic engineering, microbial cell factories, sustainable biotechnology, synthetic biology

Sustainable synthesis of chemicals, pharmaceuticals, fuels, and other value-added products has become an urgent priority among major industrial manufacturing sectors. Microorganisms have been responsible for breakthroughs in the field of medicine, industry, and bio-sustainability for thousands of years. Microbial cells serve as ideal factories for the sustainable synthesis of key economic drivers for nations. Advancements have evolved from optimizing biosynthesis capability in wild-type/natural microbial hosts to rational design and harnessing engineered microbial cell factories. Metabolic Engineering and Synthetic Biology have emerged as central disciplines of biomanufacturing, enabling the precise rewiring of metabolic pathways and engineering of microbial cells to boost titer, rate, and yield. The integration of genomic data mining, synthetic biology, and metabolic engineering has made it possible to utilize microbial systems as tools that can be programmed to produce high-quality bioactive molecules. Such an approach plays a vital role in tackling the growing problems associated with resistance to antibiotics, resource depletion, pollution and sustainable manufacturing practices. A large proportion of the biosynthesis potential of microbes is unknown, and current production processes are often limited by their capacity in terms of productivity, efficiency, and scaling. Current advances in biotechnology involve the use of new technologies like multi-omics methods, strain engineering, and innovative bioprocessing technologies to unlock untapped metabolic potential and increase functionality. The translational impact of microbiology research has also increased, including both bioprospecting of bioactive gene clusters and metabolic engineering. Though this domain has paved an avenue for next-generation biomanufacturing, it comes with its own challenges, such as metabolic bottlenecks, limited host compatibility, non-availability of genetic toolkits for non-model organisms, and lack of high-throughput screening of mutants, etc. However, current trends have seen a rapid rise in novel tools, techniques, and strategies in the field of metabolic engineering and synthetic biology. As part of this trend, this Research Topic contains articles addressing various aspects of microbial biotechnology, including the discovery of new bioactive compounds, metabolic engineering, environmental management, and pharmaceutical applications.

**Figure 1:**
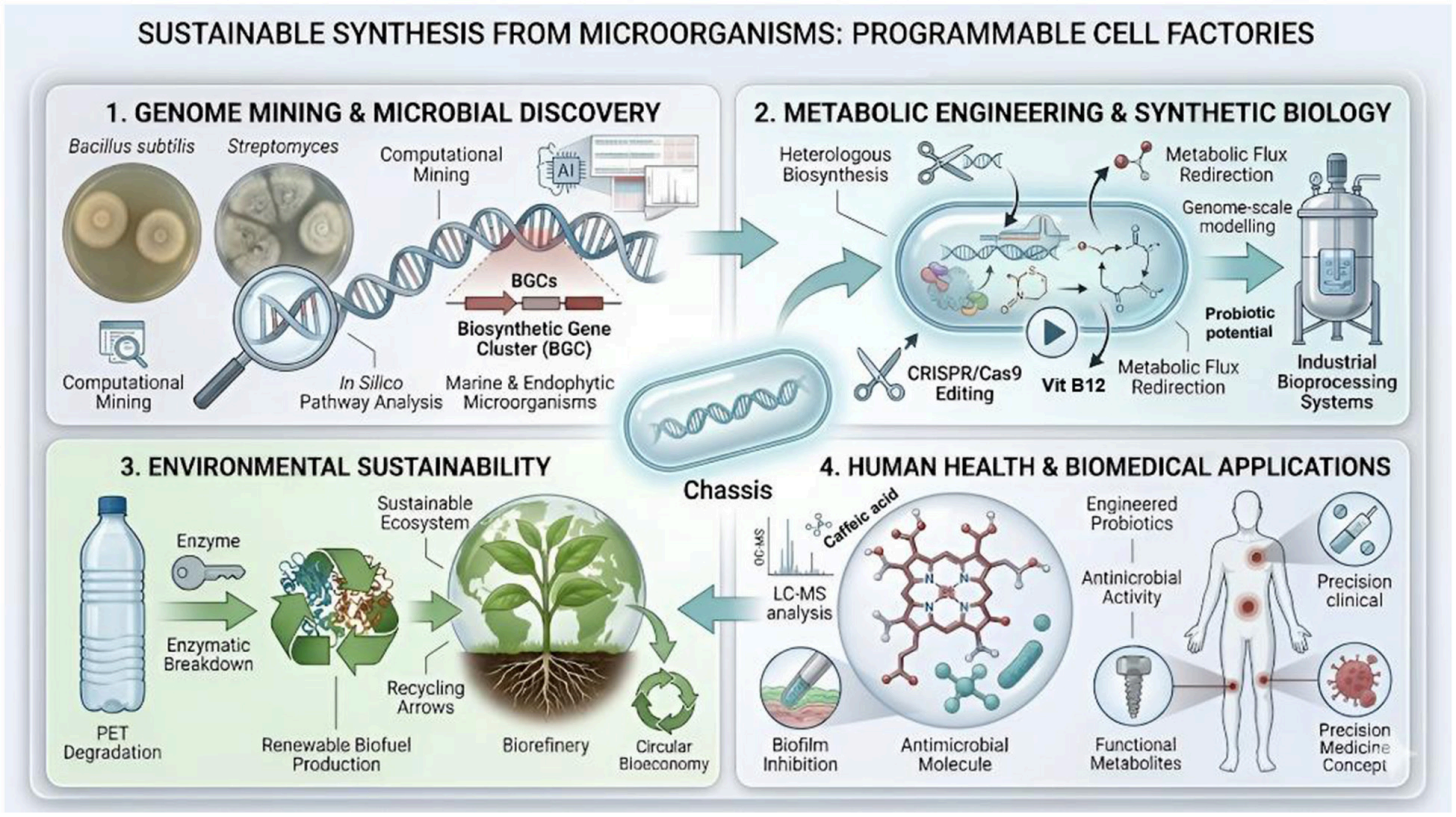
Schematic representation of the scope and applications of microbial cell factories.

The purpose of the Research Topic was to examine the emerging field of microorganisms as flexible tools in the search for bioactive substances, biomanufacturing processes, environmental purification technologies, and new therapies. This Research Topic intends to compile studies focused on the use of genome mining, metabolic engineering, microbial cell factories, and applied microbiology to explore a modern microbiological approach to addressing important world problems. Figure 1 summarizes the scope of this Research Topic and highlights the major applications of microbial cell factories in sustainable biotechnology. In total, 10 papers, among which original and review articles were compiled to highlight recent progress in the study of the biosynthetic potential of microorganisms, optimization of metabolism, and application of microbial capabilities in practice.

The current status of genome mining is such that there have been discoveries of previously unknown, cryptic biosynthetic gene clusters (BGCs) that produce metabolites with a wide range of structural diversity and biological activity. In this regard, Pattapulavar et al. have addressed microbial genomes as the major source of unexplored biosynthetic capabilities. As such, BGCs within *Streptomyces* sp. were analyzed using *in silico* tools for subsequent identification and confirmation of the activity of bioactive compounds that may serve as a promising base for next-generation antimicrobials. In line with this viewpoint, investigations carried out among endophytic and marine microbes show their potential as rich producers of novel secondary metabolites due to the particular environment they occupy.

Aside from discoveries, there are several advancements associated with the rational design and engineering of microbial cells for efficient biosynthesis of valuable chemicals. In one study, Ma et al. showed an interesting method of metabolic engineering and how the modification of tRNA m7G can help reprogram cellular metabolism to improve terpenoid biosynthesis in yeasts. Meanwhile, in a review conducted by Li et al. the synthesis of caffeic acid through heterologous production was described. This review highlighted strategies such as pathway optimization and host selection techniques to efficiently increase titers in microbes. In another study, Lin et al. improved microbial biosynthesis by designing *B. subtilis* that can effectively alter the molecular weight of the polymer to increase the versatility of poly-γ-glutamic acid in industries.

Microbial Biotechnology can also be recognized as one of the most effective tools to solve environmental problems, along with creating a bioeconomy cycle. The advances in microbial and enzymatic degradation of polyethylene terephthalate (PET) and the contribution of enzyme engineering, synthetic biology, and microbial communities in the process of depolymerization and recycling of plastics into useful products have been reviewed by Liu et al. Furthermore, the oleaginous strains of *Streptomyces* were found to be able to transform the sewage sludge into biolipids, which, in turn, were used to produce biofuels. Thus, the mentioned findings prove that the technology of microbial biotechnology provides an opportunity not only to cope with environmental problems but also to use the available resources for generating renewable energy.

Furthermore, there is evidence that the importance of using microorganisms for applications related to human health is growing, including those in the fields of biomedicine and biotechnology. Wang et al. showed that engineered microbes could serve as delivery vectors for immunotoxins for targeted cancer treatment, illustrating the ability to exploit microorganisms' specificity and genetically engineered systems to achieve enhanced therapeutic effects while avoiding shortcomings inherent to conventional approaches. On the other hand, Vijayaganapathi and Mohanasrinivasan evaluated the features of a new probiotic *Lactiplantibacillus plantarum* strain that had strong antimicrobial properties, making it suitable for use in functional products. Interestingly, Venkatesan et al. reported the Vitamin B_12_ production ability of a novel extremophile, *Ectopseudomonas alcaliphila*, along with its functional characterization. Although several organisms have been reported to synthesize B_12_ naturally, the functional characteristics of the produced B_12_ have rarely been evaluated, thereby distinguishing the biologically active form of B_12_ from pseudo-B_12_ forms. A multi-level framework combining LC-MS, *in vitro* enzyme assay, *in silico* molecular docking, and *in vivo* metabolic validation demonstrated that a novel extremophilic strain produces biologically active forms of B_12_ such as AdoCbl and MeCbl. Moreover, it has positioned the extremophilic strain as a promising microbial chassis for biochemical production. This study laid the foundation for harnessing metabolic engineering and synthetic biology as a high-throughput platform for analytical characterization of metabolites.

Overall, the research papers presented in this Research Topic provide significant insight into the revolutionary use of microorganisms as flexible and programmable systems for novel bioactive discovery, tailored biosynthesis, environmental restoration, and advanced therapeutic applications. These studies demonstrate an evident move toward systems-level microbiology that ranges from genomic mining to identify the untapped metabolic diversity of microbes to advanced engineering techniques to enable controlled and scalable microbial synthesis. Additionally, the use of microbes to solve pressing global issues, such as antibiotic resistance, pollution of the environment with plastics, waste conversion, and disease-specific treatment, is indicative of the importance of microbes beyond fundamental science. Nonetheless, some obstacles still exist despite advancements in the field, including the induction of dormant metabolic pathways, flux optimization, biosafety, and scaling up innovations from lab-based experiments to industry and clinical practice. As more researchers turn to synthetic biology and artificial intelligence-assisted design, as well as multi-omics data integration, in the coming years, the importance of microbial systems in developing sustainable and precision-driven next-generation biotechnologies cannot be overstated.

